# Supplementary material for: Nutrient restriction synergizes with retinoic acid to induce mammalian meiotic initiation in vitro
Source: Nat Commun. 2021 Mar 19;12:1758. doi: 10.1038/s41467-021-22021-6 (PMC7979727; doi:10.1038/s41467-021-22021-6)
Supplement: Supplementary file 10 — Description of Additional Supplementary Files [file 41467_2021_22021_MOESM10_ESM.pdf]

Supplementary Data 1.
Description: Genes regulated by normal medium, RA treatment, NR treatment, NRRA treatment detected by RNA-seq.

Supplementary Data 2.
Description: GO terms for clusters 1 to 4 in RNA-seq.

Supplementary Data 3.
Description: GSVA analysis.

Supplementary Data 4.
Description: Clustering information for the 165 meiosis genes.

Supplementary Data 5.
Description: GO terms for Cluster 0 to 3 in scRNA-seq.

Supplementary Data 6.
Description: Clustering information for RNA-seq analysis in WT, *Stra8*-deficient, and *Spo11*-deficient SSC cultures under normal medium and NRRA treatments.

Supplementary Data 7.
Description: Differentially expressed genes in WT and *Stra8*-deficient SSC cultures.
