# Supplementary material for: Nutrient restriction synergizes with retinoic acid to induce mammalian meiotic initiation in vitro
Source: Nat Commun. 2021 Mar 19;12:1758. doi: 10.1038/s41467-021-22021-6 (PMC7979727; doi:10.1038/s41467-021-22021-6)
Supplement: Supplementary file 11 — Reporting Summary [file 41467_2021_22021_MOESM11_ESM.pdf]

nature research

Corresponding author(s): Ning Wang

Last updated by author(s): Feb 16, 2021

Reporting Summary

Nature Research wishes to improve the reproducibility of the work that we publish. This form provides structure for consistency and transparency in reporting. For further information on Nature Research policies, see our Editorial Policies and the Editorial Policy Checklist.

Statistics

For all statistical analyses, confirm that the following items are present in the figure legend, table legend, main text, or Methods section.

n/a	Confirmed	
☐	☒	The exact sample size (*n*) for each experimental group/condition, given as a discrete number and unit of measurement
☐	☒	A statement on whether measurements were taken from distinct samples or whether the same sample was measured repeatedly
☐	☒	The statistical test(s) used AND whether they are one- or two-sided<br>*Only common tests should be described solely by name; describe more complex techniques in the Methods section.*
☒	☐	A description of all covariates tested
☐	☒	A description of any assumptions or corrections, such as tests of normality and adjustment for multiple comparisons
☐	☒	A full description of the statistical parameters including central tendency (e.g. means) or other basic estimates (e.g. regression coefficient) AND variation (e.g. standard deviation) or associated estimates of uncertainty (e.g. confidence intervals)
☐	☒	For null hypothesis testing, the test statistic (e.g. *F*, *t*, *r*) with confidence intervals, effect sizes, degrees of freedom and *P* value noted<br>*Give P values as exact values whenever suitable.*
☒	☐	For Bayesian analysis, information on the choice of priors and Markov chain Monte Carlo settings
☒	☐	For hierarchical and complex designs, identification of the appropriate level for tests and full reporting of outcomes
☐	☒	Estimates of effect sizes (e.g. Cohen's *d*, Pearson's *r*), indicating how they were calculated

Our web collection on statistics for biologists contains articles on many of the points above.

Software and code

Policy information about availability of computer code

Data collection

scRNA-seq UMI counts were estimated from the raw data using the Cell Ranger v.1.3. Confocal images were captured using Nikon NIS Elements (version 5.21). QuantStudio Software was used for qPCR data collection.

Data analysis

Images were processed using Nikon NIS Elements (version 5.21) and Adobe Photoshop 2021.

All data analyses were done using custome R code:
STAR (v.2.5.2b) was used for read mapping.
Sequence data is converted from .bcl file format to fastq file format using bcl2fastq software.
Pheatmap R packages was used to plot heatmaps.
ggplot2 R package (v3.1.0) was used for data visualization.
Cell Ranger(v1.13) was used to generate the single-cell expression raw counts.
Seurat R package (v3.1) was mainly used to analyze the scRNA-seq data, in addition to some custom R code.
Monocle package (v2.10.0) was used for pseudotime construction.
The R/Bioconductor sva package (3.30.1) was used to remove batch effect between scRNA-seq and bulk RNA-seq data.
Differentially expressed genes (DEGs) were calculated by DEseq2.
Trajectories were calculated by using the diffusion map results as an input data and using the Slingshot R package.
Gene Ontology (GO) analysis of genes were analyzed by clusterProfiler package.
GSVA heatmap was generated by R package GSVA.

For manuscripts utilizing custom algorithms or software that are central to the research but not yet described in published literature, software must be made available to editors and reviewers. We strongly encourage code deposition in a community repository (e.g. GitHub). See the Nature Research guidelines for submitting code & software for further information.

Data

Policy information about availability of data

All manuscripts must include a data availability statement. This statement should provide the following information, where applicable:

- Accession codes, unique identifiers, or web links for publicly available datasets
- A list of figures that have associated raw data
- A description of any restrictions on data availability

The authors declare that all data supporting the findings of this study are available within the article and its supplementary information files or from the corresponding author upon reasonable request. FastQ files of RNA-seq and single-cell RNA-seq are available on Gene Expression Omnibus (GEO) database under accession code: GSE153300. Files used to generate the results are available on Zenodo: https://doi.org/10.5281/zenodo.4535405.

Field-specific reporting

Please select the one below that is the best fit for your research. If you are not sure, read the appropriate sections before making your selection.

☒ Life sciences ☐ Behavioural & social sciences ☐ Ecological, evolutionary & environmental sciences

For a reference copy of the document with all sections, see nature.com/documents/nr-reporting-summary-flat.pdf

Life sciences study design

All studies must disclose on these points even when the disclosure is negative.

Sample size	Sample size calculation was not performed in this study. Sample size was determined based on previous studies and literatures, e.g., EMBO J (2017) 36:3100-3119, Nat Cell Biol (2019) 21:835-844. Sample sizes are detailed in the figure legends.
Data exclusions	Quality filtering of single-cell transcriptional profile follows standard procedure (https://satijalab.org/seurat/articles/pbmc3k_tutorial.html) and is explicitly described in Method.
Replication	All the sequencing experiments includes two independent biological replicates; all of other experiments includes at least two or three biological replicates and were repeated independently at least three times with reproducible results.
Randomization	Animals were randomized into the experimental groups. For cell culture experiments, randomization was not used, because cells were maintained and handled in the same way by the same researcher regardless of the treatments.
Blinding	No blinding was used, because genotyping for mice and cell culture treatment are needed to be conducted by the researcher.

Reporting for specific materials, systems and methods

We require information from authors about some types of materials, experimental systems and methods used in many studies. Here, indicate whether each material, system or method listed is relevant to your study. If you are not sure if a list item applies to your research, read the appropriate section before selecting a response.

Materials & experimental systems

n/a	Involved in the study
☐	☒ Antibodies
☐	☒ Eukaryotic cell lines
☒	☐ Palaeontology and archaeology
☐	☒ Animals and other organisms
☒	☐ Human research participants
☒	☐ Clinical data
☒	☐ Dual use research of concern

Methods

n/a	Involved in the study
☒	☐ ChIP-seq
☒	☐ Flow cytometry
☒	☐ MRI-based neuroimaging

Antibodies

Antibodies used

DMC1 Sigma HPA001232 diluted 1:100
SYCP3 Santa Cruz Biotechnology Sc-74569 diluted 1:400
SPATA22 Proteintech 16989-1-AP diluted 1:100
MEIOB Gift from Jeremy Wang lab diluted 1:100
RAD51 Millipore PC-130 diluted 1:200
γH2AX Millipore 05-636 diluted 1:800
DDX4 Abcam Ab13840 diluted 1:500
GFRA1 R&D AF560 diluted 1:200

	CDH1 BD 610181 diluted 1:800<br>Secondary donkey anti-mouse, anti-rabbit or anti-goat antibodies conjugated to AlexaFluor-488, AlexaFluor-546 or AlexaFluor-647 were purchased from Thermo Fisher and used at 1:500 dilution.
Validation	All of the antibodies used in this study are commercially available and validated for the application and species by the manufacturers, whose data are described at the manufacturer's website.<br><br>DMC1 Sigma HPA001232 (validation: DOI: 10.1016/s1097-2765(04)00218-7)<br>SYCP3 Santa Cruz BioSc-74569 (validation: DOI: 10.1126/sciadv.abb1660)<br>SPATA22 Proteintech 16989-1-AP (validation: DOI: 10.1038/ncomms3788)<br>MEIOB Gift from Jeremy Wang (validation: DOI: 10.1038/ncomms3788)<br>RAD51 Millipore PC-130 (validation: DOI: 10.1242/jcs.046706)<br>γH2AX Millipore 05-636 (validation: DOI: 10.1038/ncomms9036)<br>DDX4 Abcam Ab13840 (validation: DOI: 10.1093/biolre/ioaa042)<br>GFRA1 R&D AF560 (validation: DOI: 10.1016/j.celrep.2019.11.100)<br>CDH1 BD 610181 (validation: DOI: 10.1016/j.chom.2020.10.002)

Eukaryotic cell lines

Policy information about cell lines

Cell line source(s)	The WT, Stra8KO, Spo11KO mouse primary spermatogonia cell cultures in C57BL/6XDBA2 F1 backgrounds were generated by the our laboratory. The mouse primary spermatogonia cell cultures in CD1 background was generated by the our laboratory.
Authentication	Validated by staining using antibodies againast marker protein expression in undifferentiated spermatogonia. RNA-seq and scRNA-seq results further confirmed global gene expression pattern that is consistent with undifferentiated spermatogonia.
Mycoplasma contamination	Tested negative by Sigma mycoplasma detection kit.
Commonly misidentified lines<br>(See ICLAC register)	None of the cell lines used in this study is listed in the database of commonly misidentified cell lines maintained by ICLAC.
